# Gametocyte prevalence and risk factors of *P. falciparum* malaria patients admitted at the Hospital for Tropical Diseases, Thailand: a 20-year retrospective study

**DOI:** 10.1186/s12936-023-04728-7

**Published:** 2023-10-23

**Authors:** Panita Looareesuwan, Srivicha Krudsood, Saranath Lawpoolsri, Noppadon Tangpukdee, Wasin Matsee, Wang Nguitragool, Polrat Wilairatana

**Affiliations:** 1 Department of Social and Environmental Medicine, Faculty of Tropical Medicine, Mahidol University, Bangkok, 10400 Thailand; 2 Thai Travel Clinic, Hospital for Tropical Diseases, Faculty of Tropical Medicine, Mahidol University, Bangkok, 10400 Thailand; 3 Department of Tropical Hygiene, Faculty of Tropical Medicine, Mahidol University, Bangkok, 10400 Thailand; 4 Clinical Malaria Research Unit, Faculty of Tropical Medicine, Mahidol University, Bangkok, 10400 Thailand; 5 Department of Clinical Tropical Medicine, Faculty of Tropical Medicine, Mahidol University, Bangkok, 10400 Thailand; 6 Mahidol Vivax Research Unit, Faculty of Tropical Medicine, Mahidol University, Bangkok, 10400 Thailand; 7 Department of Molecular Tropical Medicine and Genetics, Faculty of Tropical Medicine, Mahidol University, Bangkok, 10400 Thailand

**Keywords:** Malaria, *Plasmodium falciparum*, Gametocyte, Risk factors

## Abstract

**Background:**

The incidence of malaria in Thailand has dramatically declined over the past two decades, and the goal is to eliminate malaria by 2025. Despite significant progress, one of the key challenges to malaria elimination are undetected gametocyte carriers. Human migration adds complexity to the malaria situation, as it not only sustains local transmission but also poses the risk of spreading drug-resistant parasites. Currently, no study has assessed the prevalence of gametocytes across multiple years in *Plasmodium falciparum* malaria patients in Thailand, and the risk factors for gametocyte carriage have not been fully explored.

**Methods:**

Medical records of all *P. falciparum* malaria patients admitted from January 1, 2001 to December 31, 2020 at the Hospital for Tropical Diseases, Thailand, were retrospectively examined and a total of 1962 records were included for analysis. Both *P. falciparum* parasites and gametocytes were diagnosed by microscopy. A regression model was used to evaluate predictors of gametocyte carriage.

**Results:**

The study demonstrated gametocyte prevalence in low malaria transmission areas. Nine risk factors for gametocyte carriage were identified: age between 15 and 24 years [adjusted odds ratio (aOR) = 1.96, 95% confidence interval (CI) 1.18−3.26], Karen ethnicity (aOR = 2.59, 95% CI 1.56−4.29), preadmission duration of fever > 7 days (aOR = 5.40, 95% CI 3.92−7.41), fever on admission (> 37.5 °C) (aOR = 0.61, 95% CI 0.48−0.77), haemoglobin ≤ 8 g/dL (aOR = 3.32, 95% CI 2.06−5.33), asexual parasite density > 5000−25,000/µL (aOR = 0.71, 95% CI 0.52−0.98), asexual parasite density > 25,000−100,000/µL (aOR = 0.74, 95% CI 0.53−1.03), asexual parasite density > 100,000/µL (aOR = 0.51, 95% CI 0.36−0.72), platelet count ≤ 100,000/µL (aOR = 0.65, 95% CI 0.50−0.85, clinical features of severe malaria (aOR = 2.33, 95% CI 1.76−3.10) and dry season (aOR = 1.41, 95% CI 1.10−1.80). An increasing incidence of imported transnational malaria cases was observed over the past two decades.

**Conclusions:**

This is the first study to determine the prevalence of gametocytes among patients with symptomatic *P. falciparum* malaria, identify the risk factors for gametocyte carriage, and potential gametocyte carriers in Thailand. Blocking transmission is one of the key strategies for eliminating malaria in these areas. The results might provide important information for targeting gametocyte carriers and improving the allocation of resources for malaria control in Thailand. This study supports the already nationally recommended use of a single dose of primaquine in symptomatic *P. falciparum* malaria patients to clear gametocytes.

**Graphical Abstract:**

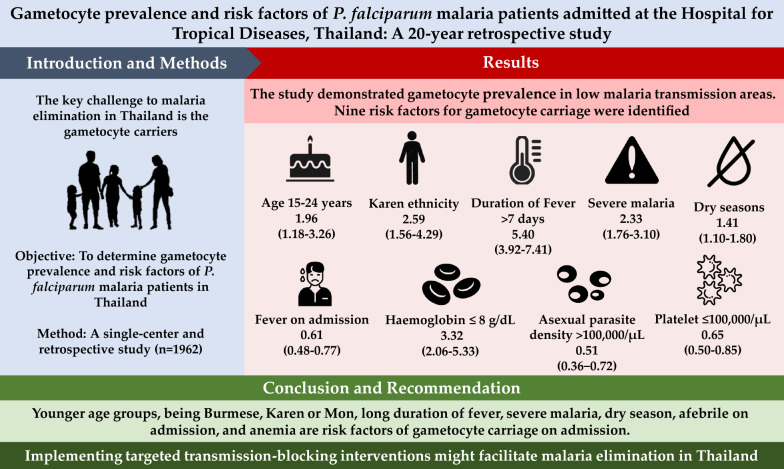

**Supplementary Information:**

The online version contains supplementary material available at 10.1186/s12936-023-04728-7.

## Background

The World Health Organization (WHO) has considered Thailand as one of the countries in the Greater Mekong Subregion (GMS) that can potentially eliminate malaria by the year 2025 [1]. While malaria has been successfully eliminated from most provinces in Thailand, transmission persists in the border regions adjacent to Myanmar and Cambodia [2, 3]. Eliminating malaria in these areas presents a significant challenge due to the complex epidemiology of malaria, such as asymptomatic malaria patients [4], multidrug-resistant parasites, population movement, geographically remote areas and political uncertainty in neighbouring countries [5].

Gametocyte carriage has posed a significant challenge to malaria control in Thailand. A recent study found that the gametocyte rate measured by qRT‒PCR was very high (72%) among asymptomatic patients infected with *Plasmodium falciparum* in communities in western Thailand [4]. As these individuals usually had no symptoms and did not seek medical treatment, they could contribute to sustained malaria transmission [4]. Cross-border movement [6] and international travel [7] have increased the risk of malaria resurgence or importation of malaria cases to malaria-free countries. Sriwichai et al*.* demonstrated that *P. falciparum* was mainly imported among Thai-Myanmar immigrants [8], and several studies have reported a higher prevalence of malaria among migrants coming from Myanmar, thus highlighting the importance of border-crossing as a factor in malaria morbidity and mortality in Thailand [9, 10]. Given that the GMS is considered the centre for drug-resistant parasites, population movement may cause the spread of artemisinin-resistant parasites to other countries [11]. In the past, isolated *P. falciparum* that was resistant to chloroquine and pyrimethamine migrated from the GMS to Africa [11, 12]. Currently, artemisinin-resistant *P. falciparum* genotypes have been identified in Africa [13, 14].

Gametocytaemia is essential for the onward transmission of malaria infection to mosquitoes, and it does not cause any clinical symptoms. The development of *P. falciparum* gametocytes occurs over a long period, unlike other *Plasmodium* species, typically at 7 to 15 days after the initial wave of asexual parasites exit into bloodstream from the liver [15]. Immature gametocytes (stages I–IV) are sequestered in the spleen and bone marrow [16]. Once the gametocytes mature, stage V gametocytes will reappear in blood with an estimated mean circulation time of 4.6–6.5 days [17]. A study in Kenyan children demonstrated the longest duration of gametocyte carriage to be 188 days [18]. In contrast, a study conducted in Thailand showed that the median gametocyte clearance time for uncomplicated *P. falciparum* malaria was 163 h (with a range of 12–806 h) following the administration of artemisinin-based combination therapy (ACT) [19].

Several risk factors were found to be associated with gametocyte carriage, including age [20–27], gender [28], blood group, sickle cell mutation (HbS) [29], malnutrition [26], duration of illness [28, 30], absence of fever [26, 28, 31], anaemia [26, 30, 32–34], thrombocytopenia [26], parasite density [26, 28], malaria severity [32] and multiplicity of infection [31, 35]. However, it is not yet clear what drives asexual stage parasites to progress into the gametocyte stage [16].

Several studies demonstrated that a single or low dose of primaquine effectively reduces malaria transmission by shortening gametocyte circulation time or reducing infectivity of gametocytes to mosquitoes [36–40]. Artemisinin derivatives act on the asexual stage and immature gametocytes, but limited effect against mature gametocytes [41]. On the other hand, primaquine is active against mature gametocytes [42]. Therefore, the WHO now recommends a single low dose of primaquine (0.25 mg/kg) to be added with ACT drugs in cases of acute *P. falciparum* infection to reduce transmission in low-intensity malaria areas such as in GMS [42]. Likewise, the Thai National Guideline Treatment for Malaria recommends a single dose of primaquine (0.5 mg/kg) or a dosage of 30 mg for individuals weighing more than 50 kg, in order to effectively clear gametocytes [43, 44].

To successfully implement a transmission-blocking strategy, it is necessary to determine the gametocyte burden in Thailand and identify the factors that predict gametocyte carriage. No previous studies have assessed the trend of gametocyte prevalence in this area; in addition, there are a limited number of research studies on gametocyte carriage risk factors. Due to the potential severity and life threatening nature of *P, falciparum* malaria, the objective of this study was to investigate the gametocyte prevalence among patients with *P. falciparum* mono-infection in Thailand. The study sought to identify risk factors and describe the epidemiology of gametocyte carriages in this population.

## Methods

### Study setting and population

The Hospital for Tropical Diseases is a university and tertiary referral hospital located in Bangkok, Thailand. Medical records of *P. falciparum* patients admitted from January 1, 2001, to December 31, 2020, were retrospectively studied. The study population included adult patients aged 15 years or older who were infected with *P. falciparum* malaria mono-infection confirmed by microscopy. All patients in the study were symptomatic, which was defined as a history of fever prior to admission. The history of fever was not limited by any specific timeframe. The duration of fever was defined as the duration of pre-admission fever. The definitions remained consistent throughout the study period. The exclusion criteria was a history of anti-malarial medication before admission for the current episode, except for malaria chemoprophylaxis. In addition, medical records with important missing data, such as malaria count charts, were excluded. Demographic data, travel details, and clinical and laboratory data were collected. The authors retrospectively reviewed medical charts and diagnosed severe malaria in accordance with the WHO criteria [45].

### Quantification of malaria parasitaemia

Giemsa-stained thick and thin films were prepared from finger-prick blood samples. Thick blood films were examined under a microscope at 100 × magnification, with 10–15 white blood cells per microscopic field considered an indicator of satisfactory thickness. The parasites were counted per 200 white blood cells in the thick film. A minimum of 200 high-power fields had to be examined before declaring the absence of malaria parasites. The number of asexual parasites and gametocytes in 1000 red blood cells was counted in the thin film, and all malaria species and stages were recorded. The parasite and gametocyte density were calculated using actual red blood cell and white blood cell counts from the automated complete blood count result. The asexual parasites and gametocytes were counted every 12 h by WHO-certified microscopists until asexual parasites were absent for three consecutive counts. These quantification methods remained unchanged throughout the study period.

Regarding treatment, severe *P. falciparum* malaria cases were prescribed parenteral artesunate followed by oral ACTs for three days. Uncomplicated *P. falciparum* malaria cases received oral ACTs or atovaquone/proguanil. Patients enrolled in drug trials received medications according to the specific trial protocol. However, since this study focuses on analysing asexual parasites and gametocytes on admission prior to the initiation of anti-malarial drugs, the treatment administered will not affect the results. In general, patients were discharged after three consecutive negative tests for asexual parasites and a demonstrated clinical improvement. Patients enrolled in drug trials were hospitalised for 28 days prior to discharge.

### Statistical analysis

Gametocyte prevalence was calculated as the proportion of patients with positive gametocytes detected by brightfield microscopy on admission. Border malaria was defined as malaria transmission that took place along land borders, whereas transnational malaria was defined as malaria acquired from a country across an international border and did not involve the border per se [46]. Continuous data were presented as means with standard deviations for normal distribution or medians with interquartile range (IQRs) for non-normal distribution. Asexual parasite and gametocyte densities underwent log_10_ transformation and were presented as geometric means per µL blood. Categorical data were presented as numbers and percentages. Crude odds ratios (ORs) or Chi-square tests were used to compare proportions as appropriate, while *t*-tests were used for continuous data. Binary logistic regression was used to evaluate the risk factors associated with gametocyte carriage on admission. Pearson’s correlation coefficient (*r*) was used to assess the linear correlation between two continuous variables. Data were analysed using SPSS version 23 (IBM Corp. in Armonk, NY). Statistical significance was set at 0.05.

## Results

A total of 2341 *P. falciparum* malaria cases were admitted to the Hospital for Tropical Diseases between 2001 and 2020. Of these, 379 records were excluded due to mixed infection, age less than 15 years old, missing laboratory data or a history of treatment with anti-malaria drugs. Thus, 1962 medical records were included for analysis.

### Demographic characteristics

Approximately 80% of patients were male and the median age was 24 years (IQR 20–31) (Table 1). The ethnicity of patients represented a very heterogeneous population that was classified into six groups: Burmese (42.9%), followed by Karen (23.4%), Mon (18.6%), Thai (11.8%), African (1.0%) and others (2.3%). Regarding occupations, 58.8%, 17.3%, 11.0%, 7.6% and 2.6% were manual labourers, unemployed, farmers, employees, and merchants, respectively. No prior history of malaria infection was reported by 50.0% of patients, while 23.5% and 19.5% of patients reported being infected with malaria either once or twice or more, respectively (Table 1). None of the patients had taken malaria chemoprophylaxis.

**Table 1:** Characteristics of *P. falciparum* patients (N = 1962)

Characteristics	*n (%)*
Gender	
Male	1562 (79.6)
Female	400 (20.4)
Median age, IQR	24, 20−31
15−24	1007 (51.3)
25−34	559 (28.5)
35−44	246 (12.5)
≥ 45	150 (7.6)
Ethnicity	
Burmese	842 (42.9)
Karen	460 (23.4)
Mon	365 (18.6)
Thai	231 (11.8)
African	20 (1.0)
Others	44 (2.3)
Occupation (N = 1955)	
Manual labourers	1150 (58.8)
Unemployed	338 (17.3)
Farmers	216 (11.0)
Employees	148 (7.6)
Merchants	50 (2.6)
Others forest-related^a^	53 (2.7)
History of malaria infection	
0	981 (50.0)
1	461 (23.5)
≥ 2	383 (19.5)
No data	137 (7.0)

*IQR* interquartile range

^a^Monk, military, woodcutters, and forest settlers

### Characteristics of *P. falciparum* malaria infection

Of 1962 patients analysed, 1352 (68.9%) had documented fever on admission. The majority of patients (1635/1962, 83.3%) had a fever duration of less than seven days, with a median duration of four days (Table 2) preadmission. There were 632 patients (32.2%) who harboured gametocytes on admission, and 173 (8.8%) showed gametocytes during treatment, which resulted in a total of 805 (41.0%) patients with gametocytes on or during admission. The median number of days to gametocyte emergence was two days (IQR 2–4). The geometric mean asexual parasite density and geometric mean gametocyte density on admission were 19,293/µL and 102/µL, respectively.

**Table 2:** Characteristics of patients with *P. falciparum* malaria infection

Characteristics	
Fever on admission, n/N (%)	1352/1962 (68.9%)
Median duration of fever, days (IQR)^a^	4 (3−5)
Gametocyte presence on admission, n/N (%)	632/1962 (32.2%)
Gametocyte presence on or during admission, n/N (%)	805/1962 (41.0%)
Gametocyte emergence during admission, days (IQR)	2 (2−4)
Geometric mean parasite density on admission, /µL (SD)	19,293 (10)
Geometric mean gametocyte density on admission, /µL (SD)	102 (5)
Mean white blood cells, 10^3^/µL (SD)	5.8 (2.5)

*IQR* interquartile range, *SD* standard deviation

^a^n = 1961

### Characteristics of *P. falciparum* malaria infection in cases with gametocytes versus no gametocytes on admission

The patients were divided into two groups upon admission: with gametocytes and without gametocytes. Independent* t*-test was used to calculate the differences in parasite characteristics between the two groups. Although the continuous data showed non-normal distribution, the sample size was sufficiently large enough for equal variances to be assumed. Two-tailed *p values* of less than 0.05 were considered statistically significant.

Regarding malaria characteristics between these two groups, three parameters were significantly different (Table 3). Patients with gametocytes on admission had a significantly longer duration of fever (*p* < 0.001), were more likely to be afebrile on admission (*p* < 0.001) and had a lower maximum geometric mean asexual parasite density during admission (*p* = 0.045).

**Table 3:** Characteristics of *P. falciparum* malaria infection in cases with gametocytes versus no gametocytes on admission

	Total	No gametocytes on admission N = 1330	Gametocytes on admission N = 632	*p value*
	Mean (SD)			
Duration of fever, days^a^	4.5 (3.3)	4.0 (2.7)	5.7 (4.0)	< 0.001
Min–Max duration of fever, days	0−36	1−36	0−30	–
Fever on admission (> 37.5 °C), N (%)^b^	1352 (68.9)	954 (71.7)	398 (63.0)	< 0.001
Geometric mean of asexual parasite density on admission, /µL	19,293 (10)	20,620 (10)	16,773 (10)	0.315
Maximum geometric mean of asexual parasite density during admission, /µL	24,071 (10)	25,840 (9)	20,730 (11)	0.045
Average time to maximum parasite density, hours	10 (3)	10 (4)	10 (3)	0.175

^a^n = 1961

^b^Chi-square test

### Number of *P. falciparum* malaria cases and gametocyte prevalence

**Fig. 1:**
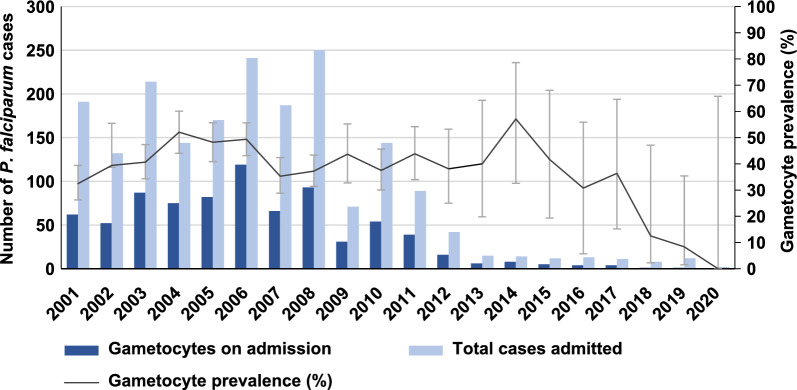
Gametocyte prevalence of *Plasmodium falciparum* malaria admitted to the Hospital for Tropical Diseases, Thailand, between 2001 and 2020

There was a marked decline in the number of *P. falciparum* malaria cases admitted in the hospital. From 2001 to 2012, the gametocyte prevalence recorded at the Hospital for Tropical Diseases, Thailand, ranged from 32 to 52%. Between 2013 and 2020, there was a decreasing trend in gametocyte prevalence (Fig. 1). However, it is important to interpret these numbers with caution due to the small sample size.

Between 2001 and 2012, the main study population was composed of Burmese, Karen, and Mon patients, whereas after 2012, there were more African patients. Thai patients were presented across the study period (Additional file 1). In the past decade, there was an increasing proportion of imported transnational malaria from Africa admitted to the hospital, from 7% (1/15) in 2013 to 92% (11/12) in 2019 (Additional file 2).

### Gametocyte carriage risk factors

To determine risk factors for gametocyte carriage, univariate and multivariable analyses were performed based on the proportion of gametocyte carriage in each analytic group (Table 4). The cut-off *p value* for univariate analysis was 0.1. The forest plots of univariate and multivariable analyses are shown in Additional files 3 and 4.

**Table 4:** Univariate and multivariable analyses of demographic, clinical, parasitological, haematological, and other risk factors for gametocyte carriage

Characteristics	Gametocytes on admission		Crude OR (95% CI)	*p* value	Adjusted OR (95% CI)
	No	Yes			
Gender (n = 1962)					–
Male	1061 (67.9)	501 (32.1)	1	0.796	
Female	269 (67.3)	131 (32.8)	1.03 (0.82−1.30)		
Age, years (n = 1962)					*p* value = 0.008
15–24	630 (62.6)	377 (37.4)	2.39 (1.57−3.64)	< 0.001	1.96 (1.18−3.26)
25–34	399 (71.4)	160 (28.6)	1.60 (1.03−2.49)		1.47 (0.87−2.50)
35–44	181 (73.6)	65 (26.4)	1.44 (0.88−2.35)		1.24 (0.69−2.21)
≥ 45	120 (80.0)	30 (20.0)	1		1
Occupation (n = 1955)					*p* value = 0.624
Employees	108 (73.0)	40 (27.0)	1	< 0.001	1
Unemployed	197 (58.3)	141 (41.7)	1.93 (1.27−2.95)		0.99 (0.62−1.60)
Manual labourers	777 (67.6)	373 (32.4)	1.30 (0.88−1.90)		0.86 (0.48−1.52)
Merchants	37 (74.0)	13 (26.0)	0.95 (0.46−1.97)		1.47 (0.59−3.62)
Farmers	163 (75.5)	53 (24.5)	0.88 (0.55−1.42)		1.11 (0.67−1.84)
Others	43 (81.1)	10 (18.9)	0.63 (0.29−1.37)		0.61 (0.26−1.47)
Ethnicity (n = 1962)					*p* value = 0.005
Thai	191 (82.7)	40 (17.3)	1	< 0.001	1
Burmese	577 (68.5)	265 (31.5)	2.19 (1.51−3.18)		2.25 (1.37−3.69)
Karen	287 (62.4)	173 (37.6)	2.88 (1.95−4.25)		2.59 (1.56−4.29)
Mon	228 (62.5)	137 (37.5)	2.87 (1.92−4.29)		2.51 (1.49−4.21)
African	19 (95.0)	1 (5.0)	0.25 (0.03−1.93)		0.19 (0.16−2.17)
Others	28 (63.6)	16 (36.4)	2.73 (1.35−5.51)		2.73 (1.17−6.40)
BMI, kg/m^2^ (n = 1943)					*p* value = 0.605
Normal $\ge$ 18.5–25	953(68.9)	430 (31.1)	1	0.06	1
Underweight < 18.5	296 (64.2)	165 (35.8)	1.24 (0.99−1.54)		0.96 (0.73−1.25)
Overweight > 25	74 (74.7)	25 (25.3)	0.75 (0.47−1.10)		1.30 (0.74−1.94)
Population group (n = 1962)^a^					–
Thai	191 (82.7)	40 (17.3)	1	< 0.001	
Immigrant	1116 (65.5)	589 (34.5)	2.52 (1.77−3.56)		
Travellers	23 (88.5)	3 (11.5)	0.62 (0.18−2.18)		
History of malaria (n = 1825)					*p* value = 0.746
None	635 (64.8)	345 (35.3)	1	0.008	1
1 time	322 (69.8)	139 (30.2)	0.79 (0.62−1.01)		1.08 (0.81−1.43)
≥ 2 times	279 (72.8)	346 (35.3)	0.68 (0.53−0.89)		0.96 (0.73−1.25)
Duration of fever, days (n = 1961)					*p* value < 0.001
≤ 3	702 (80.6)	169 (19.4)	1	< 0.001	1
> 3–7	487 (63.7)	277 (36.3)	2.36 (1.89−2.95)		2.47 (1.91−3.19)
> 7	141 (43.3)	185 (56.7)	5.45 (4.14−7.12)		5.40 (3.92−7.41)
Fever on admission (> 37.5 °C) (n = 1962)					*p* value < 0.001
No	376 (61.6)	234 (38.4)	1	< 0.001	1
Yes	954 (70.6)	398 (29.4)	0.67 (0.55−0.82)		0.61 (0.48−0.77)
Asexual parasite density, /µL (n = 1962)					*p* value = 0.002
≤ 5000	290 (62.2)	176 (37.8)	1	0.017	1
> 5000–25,000	334 (67.5)	161 (32.5)	0.79 (0.61−1.04)		0.71 (0.52−0.98)
> 25,000–100,000	346 (69.9)	149 (30.1)	0.71 (0.54−0.93)		0.74 (0.53−1.03)
> 100,000	360 (71.1)	146 (32.2)	0.67 (0.51−0.87)		0.51 (0.36−0.72)
Haemoglobin (≤ 8 g/dL) (n = 1961)					*p* value < 0.001
No	1298 (70.9)	532 (29.1)	1	< 0.001	1
Yes	31 (23.7)	100 (76.3)	7.87 (5.20−11.92)		3.32 (2.06−5.33)
Thrombocytopenia, (≤ 100,000/µL) (n = 1962)					*p* value = 0.002
No	435 (63.9)	246 (36.1)	1	0.007	1
Yes	894 (69.8)	386 (30.2)	0.76 (0.63−0.93)		0.65 (0.50−0.85)
Severe malaria					*p* value < 0.001
No	1051 (73.3)	383 (26.7)	1	< 0.001	1
Yes	279 (52.8)	249 (47.2)	2.45 (1.99–3.01)		2.33 (1.76−3.10)
Seasonality^b^					*p* value = 0.006
Rainy	925 (69.3)	409 (30.7)	1	0.032	1
Dry	405 (64.5)	223 (35.5)	1.25 (1.02–1.52)		1.41 (1.10−1.79)
Import malaria (n = 1934)					*p* value = 0.128
Indigenous	673 (71.5)	268 (28.5)	1	< 0.001	1
Border	595 (62.6)	356 (37.4)	1.50 (1.24−1.82)		1.28 (1.01−1.62)
Transnational	37 (88.1)	5 (11.9)	0.34 (0.13−0.87)		1.07 (0.28−4.07)

^a^Population group was not included in the multivariable logistic regression model due to collinearity with ethnicity

Immigrant refers to patients whose ethnicities were Burmese, Cambodian, Laotian and minor ethnicities. Travellers refer to patients whose ethnicities were neither Thai nor immigrant

^b^Rainy season between May and October, dry season between September and April

### Demographic risk factors

Univariate analysis revealed five demographic risk factors for gametocytaemia on admission: age, occupation, ethnicity, body mass index (BMI) and population group. The younger age group of 15–24 years (OR = 2.39, 95% confidence interval (CI) 1.57–3.64, *p* < 0.001) had higher odds of gametocytaemia compared with the older age groups. Regarding occupation, those who were unemployed had the highest risk of carrying gametocytes compared to other occupations (OR = 1.93, 95% CI 1.27–2.95, *p* < 0.001). Karen, Mon and Burmese ethnicities demonstrated two to three times higher the odds of gametocytaemia than Thai citizens. Underweight individuals (BMI < 18.5 kg/m^2^) (OR = 1.24, 95% CI 0.99–1.54, *p* = 0.06) and immigrants were risk factors for gametocyte carriage on admission.

Although not statistically significant, African ethnicities (OR = 0.25, 95% CI 0.03–1.93), overweight individuals (BMI > 25 kg/m^2^) (OR = 0.75, 95% CI 0.47−1.10) and travellers (OR = 0.62, 95% CI 0.18−2.18 were associated with a low risk of gametocyte carriage on admission. No significant association was observed between gametocyte carriage and gender.

### Clinical, parasitological, and haematological risk factors

Gametocyte carriage was significantly associated with a fever duration > 3 days (OR = 3.06, 95% CI 2.49 − 3.76, *p* < 0.001), haemoglobin ≤ 8 g/dL (OR = 7.87, 95% CI 5.20−11.92, *p* < 0.001) and severe malaria (OR = 2.45, 95% CI 1.99−3.01, *p* < 0.001). However, the correlation between fever duration and gametocyte density was weak, despite a statistically significant *p* value (Pearson’s correlation coefficient = 0.113, *p* < 0.004, Additional file 5). In contrast, gametocyte carriage decreased with a history of malaria infection (OR = 0.79, 95% CI 0.62−1.01, *p* = 0.08), fever on admission (axillary temperature (> 37.5 °C) (OR = 0.67, 95% CI 0.55−0.82, *p* < 0.001), asexual parasite density > 5000−25,000/µL (OR = 0.79, 95% CI 0.61−1.04), asexual parasite density > 25,000−100,000/µL (OR = 0.74, 95% CI 0.54−0.93), asexual parasite density > 100,000/µL (OR = 0.67, 95% CI 0.51−0.87), and thrombocytopenia (≤ 100,000/µL) (OR = 0.76, 95% CI 0.63−0.93, *p* = 0.007). There was no significant correlation between gametocyte density and asexual parasite density among different ethnicities (Additional file 6).

### Seasonality and cases of imported *P. falciparum* malaria

In Thailand, the rainy season is between May and October, and the dry season is between September and April [47]. A higher number of *P. falciparum* malaria cases were observed during the rainy season (Additional file 7). The proportion of patients with positive gametocytes on admission ranged from 25% (33/130 in October) to 41% (49/119 in January and 30/74 in February). Individuals were more likely to develop gametocytes during the dry season (OR = 1.24, 95% CI 1.02−1.52, *p* = 0.032). The risk of gametocyte carriage was approximately 1.5 times higher in cases of border malaria than cases of indigenous malaria (OR = 1.5, 95% CI 1.24−1.82, *p* < 0.001) whereas transnational malaria showed a low risk of gametocyte carriage (OR = 0.34, 95% CI 0.13−0.87, *p* < 0.001).

## Multivariable analysis

All factors except gender and population group were included in the multivariable analysis. The reason was that gender showed a non-significant result (p > 0.1) and the population group had collinearity with ethnicity. Multivariable analysis revealed nine factors that had significantly higher risks of carrying gametocytes: (1) age between 15 and 24 years old, (2) being Burmese, Karen or Mon, (3) fever duration > 3 days, (4) afebrile on admission (≤ 37.5 °C), (5) haemoglobin ≤ 8 g/dL, (6) asexual parasite density ≤ 5000/µL (7) platelet count > 100,000/µL (8) clinical features of severe malaria and (9) dry season. There was no statistically significant difference in gametocyte carriage among occupations, BMI, a history of malaria and imported malaria (Table 4).

## Discussion

This study aimed to demonstrate the gametocyte prevalence of *P. falciparum* in Thailand from a 20-year retrospective study and thus identify potential risk factors that enable target malaria carriers. This is the longest retrospective study that demonstrated gametocyte prevalence in Thailand, which is a low-intensity malaria transmission area. The study demonstrated a gametocyte prevalence of 32−52% between 2001 and 2012. A relatively higher gametocyte prevalence was observed in comparison to a study at the Thai-Myanmar border during 2001−2010 (24−40% vs. 3−12%) [48]. Likewise, other earlier studies conducted in Thailand also showed a lower gametocyte prevalence [19, 30, 48]. The observed differences might be due to a longer duration of fever or more severe cases in this study population, as the study site was a malaria referral hospital. Moreover, the study identified a slightly higher gametocyte density compared to Nigerian paediatric patients [49], possibly attributed to the higher malaria endemicity in sub-Saharan Africa. The presence of acquired immunity to malaria [50] might contribute to a decrease in gametocyte density. In contrast, Thailand’s border area is recognized for its unstable and low malaria transmission [51]. Additionally, African patients might be more accustomed to malaria and tend to seek care earlier when they are ill.

Nine independent factors were identified, showing a statistically significant association with gametocytaemia on admission. A younger age group (15–24 years) was an important predictor of gametocyte carriage in the study population. Many studies have associated young age with a higher risk of gametocytaemia [21, 23–26]. A recent meta-analysis of clinical trials conducted in Asia, Africa and South America involving nearly 50,000 patients, revealed interesting findings regarding the prevalence of gametocytaemia [26]. In Africa, the prevalence of gametocytaemia decreased with age, whereas in Asia, it showed an increasing trend until approximately 20 years of age, followed by a gradual decline thereafter [26]. The lower prevalence of gametocytaemia in adults compared to children can be partly attributed to the consequences of increasing immunity against asexual parasite, as well as the possibility that the immune response directly influence gametocytogenesis [15]. A recent study identified antibodies targeting *P. falciparum* gametocytes, namely Pfs48/45 and Pfs230 [52]. However, there are limited data available regarding the age-related increase in sexual stage immunity. In contrast, some studies have reported no significant association between age and gametocyte carriage [31, 53]. The differences may be attributed to variations in population profiles and methods used for parasite detection.

Burmese migrants [8, 9] or Karen ethnicity [54] were reported as more likely to be infected with *P. falciparum* malaria than Thai citizens. This is the first study to demonstrate the association between ethnicity and gametocyte carriage. The odds of gametocytaemia in Karen, Mon, and Burmese individuals were two to three times higher than in Thai citizens. Ethnicity may not be directly associated with gametocyte development but rather related to the ecology and socio-economic structure of these populations. Difficulty in accessing healthcare system, including low-risk perception, language barriers, geographical remoteness, poor logistics and limited health coverage may lead to delayed treatment, placing this subpopulation at risk for developing gametocytes. Thus, to reduce transmission and help contain the spread of artemisinin-resistant *P. falciparum* malaria, it is imperative to administer a single dose of primaquine as a gametocytocidal drug, particularly to migrants who were identified with higher gametocyte prevalence.

Unemployed status was shown to be a risk factor for gametocytaemia in the univariate analysis but not in the multivariable analysis. Political and economic crises might have driven these individuals to migrate cross-border in search for work [8]. It has been demonstrated that recent migrants were almost four times more likely to be infected with *P. falciparum* malaria compared with Thai patients, suggesting that most *P. falciparum* cases were imported [8]. Therefore, border screening, regional collaboration, early case detection and treatment in this mobile group are crucial to prevent imported malaria [55]. Interestingly, forest-related occupations were unexpectedly not identified as a risk factor, but this could be due to the small sample size. Nevertheless, multivariable analysis did not show occupation as a risk factor for gametocyte carriage.

There were discrepancies in outcomes between studies as to whether gender [23, 28, 31], absence of fever [26, 28, 31], and parasite density [26, 28, 32] were risk factors of gametocyte carriage. Consistent with previous findings, this study demonstrated that patients who were afebrile (≤ 37.5 °C) [26, 28], had a long duration of fever > 3 days, had anaemia (haemoglobin ≤ 8 g/dL) and had asexual parasite densities ≤ 5000/μL were predictive of gametocyte carriage [28]. A longer duration of fever was found to be associated with a higher risk of gametocytaemia and an increase in gametocyte density. Therefore, in settings with limited health care facilities, a history of a long duration of fever should prompt timely diagnosis and treatment. It has also been reported that anaemia may reflect a prolonged infection and erythrocyte destruction, as evidence by the red blood cell lysis, dyserythropoiesis and reticulocytosis may trigger gametocytogenesis [56, 57]. A direct correlation between asexual parasite density and gametocyte density has also been demonstrated [31]. However, the finding could not be replicated, and this may be attributed to differences in duration of fever before admission or variations in patients’ clinical severity. Despite this, the study revealed low asexual parasite density (≤ 5000/μL) as a predictive factor of gametocytaemia, which is consistent with the literature [28]. According to Farid et al., gametocytogenesis occurred at a very low parasite density, specifically < 100 parasites per mL [58]. Cases with lower asexual parasite densities were reported as more likely to have gametocytes, suggesting that excessive multiplication compensated for reduced gametocytogenesis [59]. Another explanation may be that patients with low parasitaemia are often asymptomatic or subclinical, leading to a delay in seeking care and the subsequent gametocyte development. The study found that gametocyte carriage was more common in severe cases than in uncomplicated malaria. It is possible that the stressful environment in the host triggers gametocytogenesis, with more asexual forms committed to the sexual stage to increase the chance of parasite survival and onward transmission. Thus, further studies on gametocyte biology are necessary to determine the cause of this finding.

A higher rate of gametocyte carriers in the dry season was demonstrated in this study. Unlike previous findings, there was no association between gametocyte prevalence and seasonality [23, 31, 53]. From an evolutionary perspective, a parasite would not benefit from investing resources in gametocyte development if there were no mosquitoes for onward transmission. However, it has been speculated that parasites might increase their gametocyte conversion rate at the beginning of the transmission season [60]. In Thailand, there was little seasonal variation from year to year between 2001 and 2011 [48]. However, after 2011, marked fluctuations in rainfalls and droughts occurred due to the effects of El Niño and La Niña [61]. Since these factors may have an impact on gametocyte prevalence, further investigation is warranted.

The use of primaquine holds great potential for reducing transmission of *P. falciparum* malaria in low-transmission settings [42]. Available data suggest that a single low dose of primaquine is safe in glucose-6-phosphate dehydrogenase (G6PD) deficient patients in Southeast Asia [62, 63]. However, the current practice of administering a single dose of primaquine to block transmission in symptomatic *P. falciparum* cases, as recommended in the Thai malaria treatment guideline, does not address the asymptomatic carriers with gametocytes who do not seek medical care. These carriers, often submicroscopic, pose a hidden threat to malaria elimination. Several studies confirmed the presence of asymptomatic and submicroscopic malaria infections in Thailand [4, 64, 65]. But limited data are available regarding the gametocytes in these individuals and their infectivity to mosquitoes in low-transmission settings [66–68]. Further research on gametocytes and malaria transmission, encompassing both symptomatic and asymptomatic cases, is necessary to develop effective strategies for mitigating malaria in this low-endemicity area.

Interestingly, an increasing proportion of imported malaria cases transnationally, mainly from the African region was found. Similar situations have occurred in China, Sri Lanka and Kyrgyzstan, where imported malaria threatened malaria resurgence and reintroduction as these countries approached the elimination phase [69]. Since Thailand is approaching the pre-elimination phase, understanding the epidemiology and situation of gametocyte carriage is crucial. Border malaria is complex and requires integrated intervention with multiple parties at local and national levels between adjacent countries. Moreover, awareness of transnational malaria, especially gametocyte carriers, will be useful in preventing onward transmission and the risk of artemisinin-resistant spread.

## Limitations

There were some limitations in the current study. First, the number of *P. falciparum* malaria cases admitted during the past several years was small due to the majority of cases being treated at the border area and the recent travel restriction from the COVID-19 pandemic. However, the decrease in the number of malaria cases reflected the real-life situation. Second, submicroscopic and asymptomatic malaria cases were not included in the study; thus, the gametocyte prevalence might be underestimated. Nevertheless, microscopy remains the standard diagnostic tool and can be applied in the field studies. Third, detailed data on the timing of past malaria infections could not be obtained due to the nature of retrospective study, so the gametocytes detected on admission could also result from recent infection. Furthermore, the precise geolocation may be obscured from the constant movement of human population. As a result, considering the history of malaria and border malaria as a risk factor for gametocytaemia should be interpreted with caution. Despite these limitations, the advantages of this study include a large sample size with comprehensive data on gametocyte carriage over multiple years.

## Conclusion

This study is the first to demonstrate gametocyte prevalence of *P. falciparum* cases and gametocyte risk factors in Thailand. The results revealed that younger age groups, non-Thai citizens (Burmese, Karen and Mon), afebrile on admission, individuals with a longer duration of fever and those with severe clinical features had at a higher risk of gametocyte carriage. Implementing targeted transmission-blocking interventions in this population might facilitate malaria elimination efforts in Thailand. Additionally, raising awareness of transnational malaria, particularly regarding gametocyte carriers, would be useful in preventing onward transmission and the risk of artemisinin-resistant spread. These findings highlight the need for the bidirectional efforts in malaria control.

### Supplementary Information

**Additional file 1: **Ethnicity of *P. falciparum* malaria cases admitted to the Hospital for Tropical Diseases, Thailand, between 2001 and 2020.

**Additional file 2: **Malaria contraction sites of *P. falciparum* malaria cases admitted to the Hospital for Tropical Diseases, Thailand, between 2001 and 2020.

**Additional file 3: **Forest plot based on univariate analysis of demographic, clinical, parasitological, haematological, and other risk factors for gametocyte carriage.

**Additional file 4: **Forest plot based on multivariable analysis of demographic, clinical, parasitological, haematological, and other risk factors for gametocyte carriage.

**Additional file 5: **Scatter plot showing the association between duration of fever and gametocyte density.

**Additional file 6: **Scatter plot showing the association between asexual parasite density and gametocyte density stratified by ethnicity.

**Additional file 7: **Gametocyte prevalence and total number of *P. falciparum *malaria cases admitted to the Hospital for Tropical Diseases, Thailand recorded monthly during 2001–2020.

## Data Availability

Data are available from the corresponding author to all interested researchers upon reasonable request.

## Abbreviations

aOR: Adjusted odds ratio
CI: Confidence interval
WHO: World Health Organization
GMS: Greater Mekong Subregion
ACT: Artemisinin-based combination therapy
IQR: Interquartile range
OR: Odds ratio
BMI: Body mass index
G6PD: Glucose-6-phosphate dehydrogenase
