# Supplementary material for: Gametocyte prevalence and risk factors of P. falciparum malaria patients admitted at the Hospital for Tropical Diseases, Thailand: a 20-year retrospective study
Source: Malar J. 2023 Oct 23;22:321. doi: 10.1186/s12936-023-04728-7 (PMC10591378; doi:10.1186/s12936-023-04728-7)
Supplement: Supplementary file 2 — Additional file 2: Malaria contraction sites of P. falciparum malaria cases admitted to the Hospital for Tropical Diseases, Thailand, between 2001 and 2020. [file 12936_2023_4728_MOESM2_ESM.docx]

**Additional File 2: Malaria contraction sites of *P. falciparum* malaria cases admitted to the Hospital for Tropical Diseases, Thailand, between 2001–2020**
