# Supplementary material for: Gametocyte prevalence and risk factors of P. falciparum malaria patients admitted at the Hospital for Tropical Diseases, Thailand: a 20-year retrospective study
Source: Malar J. 2023 Oct 23;22:321. doi: 10.1186/s12936-023-04728-7 (PMC10591378; doi:10.1186/s12936-023-04728-7)
Supplement: Supplementary file 4 — Additional file 4: Forest plot based on multivariable analysis of demographic, clinical, parasitological, haematological, and other risk factors for gametocyte carriage. [file 12936_2023_4728_MOESM4_ESM.docx]

**Additional File 4: Forest plot based on multivariable analysis of demographic, clinical, parasitological, haematological, and other risk factors for gametocyte carriage**

^†^ Rainy season between May and October, dry season between September and April

BMI <18.5 kg/m^2^ - Underweight, BMI 18.5-25 kg/m^2^ - Normal weight, BMI > 25 kg/m^2^ - Overweight
