# Supplementary material for: Gametocyte prevalence and risk factors of P. falciparum malaria patients admitted at the Hospital for Tropical Diseases, Thailand: a 20-year retrospective study
Source: Malar J. 2023 Oct 23;22:321. doi: 10.1186/s12936-023-04728-7 (PMC10591378; doi:10.1186/s12936-023-04728-7)
Supplement: Supplementary file 5 — Additional file 5: Scatter plot showing the association between duration of fever and gametocyte density. [file 12936_2023_4728_MOESM5_ESM.docx]

**Additional File 5: Scatter plot showing the association between duration of fever and gametocyte density**


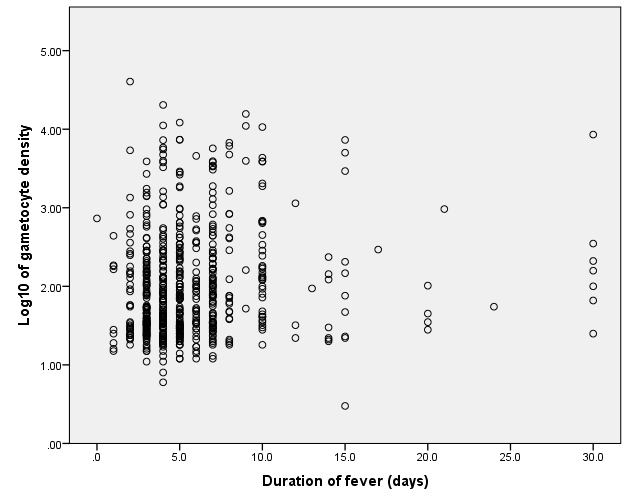


Pearson’s correlation coefficient (*r*) = 0.113, *p* value < 0.004
