# Supplementary material for: Gametocyte prevalence and risk factors of P. falciparum malaria patients admitted at the Hospital for Tropical Diseases, Thailand: a 20-year retrospective study
Source: Malar J. 2023 Oct 23;22:321. doi: 10.1186/s12936-023-04728-7 (PMC10591378; doi:10.1186/s12936-023-04728-7)
Supplement: Supplementary file 6 — Additional file 6: Scatter plot showing the association between asexual parasite density and gametocyte density stratified by ethnicity. [file 12936_2023_4728_MOESM6_ESM.docx]

**Additional File 6: Scatter plot showing the association between asexual parasite density and gametocyte density stratified by ethnicity**


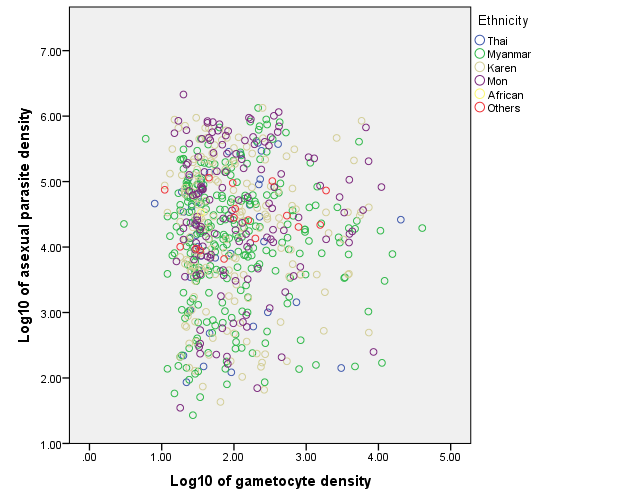


| Ethnicity | Pearson’s correlation coefficient (*r*) | *p* value |
| --- | --- | --- |
| Thai | 0.011 | 0.947 |
| Myanmar | 0.013 | 0.827 |
| Karen | -0.037 | 0.627 |
| Mon | 0.019 | 0.824 |
| Others | 0.183 | 0.498 |
| Total | 0.011 | 0.786 |

^*^ Pearson’s correlation of African ethnicity cannot be determined due to the small sample size
